# Increased Efficiency in Small Molecule Organic Solar Cells Through the Use of a 56-π Electron Acceptor – Methano Indene Fullerene

**DOI:** 10.1038/srep08319

**Published:** 2015-02-09

**Authors:** James W. Ryan, Yutaka Matsuo

**Affiliations:** 1 Department of Chemistry, School of Science, The University of Tokyo, 7-3-1 Hongo, Bunkyo-ku, Tokyo 113-0033, Japan

## Abstract

Organic solar cells (OSCs) offer the possibility of harnessing the sun's ubiquitous energy in a low-cost, environmentally friendly and renewable manner. OSCs based on small molecule semiconductors (SMOSCs) – have made a substantial improvement in recent years and are now achieving power conversion efficiencies (PCEs) that match those achieved for polymer:fullerene OSCs. To date, all efficient SMOSCs have relied on the same fullerene acceptor, PCBM, in order to achieve high performance. The use of PCBM however, is unfavourable due to its low lying LUMO level, which limits the open-circuit voltage (*V*_OC_). Alternative fullerene derivatives with higher lying LUMOs are thus required to improve the *V*_OC_. The challenge, however, is to prevent the typical concomitant decrease in the short circuit current density (*J*_SC_) when using a higher LUMO fullerene. In this communication, we address the issue by applying methano indene fullerene, MIF, a bis-functionalised C_60_ fullerene that has a LUMO level 140 mV higher than PCBM, in solution processed SMOSCs with a well known small molecule donor, DPP(TBFu)_2_. MIF-based devices show an improved *V*_OC_ of 140 mV over PC_61_BM and only a small decrease in the *J*_SC_, with the PCE increasing to 5.1% (*vs.* 4.5% for PC_61_BM).

Organic solar cells (OSCs) are becoming an increasingly viable low-cost, environmentally friendly and efficient means of generating electricity, with power conversion efficiencies exceeding 9% for single junction devices and 10% for tandem devices^1^,^2^. Within the past five years, small molecule OSCs (SMOSCs), where both the donor and acceptor are low molecular weight organic molecules, have made great strides with efficiencies now on par with their polymer:fullerene counterparts^3^,^4^. The improved performances obtained for SMOSCs has mainly come from the design of improved donor molecules that give higher absorption coefficients, better matching of the solar spectrum and enhanced miscibility with the fullerene acceptor, the latter previously being a strong limiting factor^5^,^6^,^7^. However, in all efficient solution processed BHJ SMOSCs to date the fullerene acceptor has been PCBM. In the case of polymer:fullerene OSCs a few examples of alternative fullerene derivatives do exist that allow higher efficiencies to be achieved by improving the open-circuit voltage (*V*_OC_)^8^,^9^. This improvement in *V*_OC_ stems from the fullerene derivatives possessing a higher lying LUMO level, as the *V*_OC_ is roughly proportional to the difference between the HOMO of the donor and LUMO of the acceptor^10^. Non-geminate recombination and the occupancy of the donor and acceptor density of states (DOS) will also have an effect on the *V*_OC_^11^,^12^,^13^,^14^, as can device architecture and the choice of selective contacts^15^,^16^.

Raising the LUMO level of a fullerene molecule can be achieved either through the addition of an electron donating moiety or by reducing the π-conjugation from 60-π electrons for C60 to 58-π electrons by the addition of a single adduct and to 56-π electrons by adding an additional adduct and so on^17^,^18^,^19^. Indene-C60 bisadduct (ICBA) is a good example of a bisadduct 56-π fullerene that improves the efficiency of P3HT based polymer:fullerene solar cells to 6.5%, which is significantly higher than the that of P3HT:PCBM, 3.8%, due to a 0.26 V increase in *V*_OC_^8^. However, except for P3HT and a few other examples^20^, most polymers blended with alternative fullerenes show significant losses in the short circuit current (*J*_SC_), which reduces or indeed negates the improvements in the overall power conversion efficiency (PCE, η) made through the increase in *V*_OC_^21^,^22^,^23^. Several explanations for this decrease in photocurrent have been presented. For example, Faist et al. found that a range of polymer donors blended with multiadduct fullerenes show low *J*_SC_ values due either to reduced charge transfer efficiencies caused by small HOMO-HOMO or LUMO-LUMO offset energies between the donor and acceptor, or to poor charge collection efficiencies^21^. In the case of donor-acceptor combinations that produce *V*_OC_ values that exceed 1 V, Hoke and co-workers found that the HOMO energy offsets between the donor and fullerene can prevent efficient hole transfer, which limits *J*_SC_^22^.

In SMOSC research, very few examples of non-PCBM acceptors exist. We previously found that, in the case of *p*-*i*-*n* devices based on a benzoporphyrin donor, higher efficiencies can be obtained using 58-π silylmethyl fullerene (SIMEF) acceptors rather than PC_61_BM, due to SIMEF having a higher lying LUMO level and thus generating a higher *V*_OC_, as well as an improved *J*_SC_ due to the better packing of SIMEF^24^,^25^. A recent study by Palomares and co-workers attempted to understand the effect of replacing PC_71_BM with C70-based diphenylmethano fullerenes (DPMs) where the alkyl chains on the phenyl groups varied between 4 and 12 carbons^26^. In this study the donor molecule was a diketopyrolopyrole derivative, DPP(TBFu)_2_, which is a highly absorbing molecule (in solution it has a molar absorptivity of 64 000 M^-1^ cm^-1^ at 630 nm) that was developed by Nguyen and co-workers^27^. The effect of changing the fullerene adduct as well as the length of the terminal alkyl chains on the DPM adduct was found to affect the growth and crystallization of DPP(TBFu)_2_ domains, which led to significantly lower *J*_SC_ values than the reference PC_71_BM devices. Although both DPM and PCBM were found to have the same LUMO level, *V*_OC_ was higher for devices employing DPM fullerenes due to slower non-geminate recombination dynamics.

Here we apply a 56-π bis-functionalised fullerene (MIF), where one adduct is an indene group and the other is methanediyl group^9^, in SMOSCs using the well known DPP(TBFu)_2_ as a donor^27^, with the aim of improving the *V*_OC_ due to the higher LUMO of this 56-π fullerene (−3.66 eV vs. 3.80 eV for PC_61_BM)^9^. The indene group provides for a good “alkyl chain-free” solubilizing group while the methanediyl adduct is the smallest possible adduct a fullerene can be functionalized with. This combination of adducts was chosen to reduce the conjugation of the fullerene in a manner that provides adequate solubility without using bulky adducts or adducts containing long alkyl chain substituents that could prevent, or at least limit, the formation of well-ordered DPP(TBFu)_2_ domains. Fig. 1 shows the device architecture and molecular structures and energy levels of DPP(TBFu)_2_ and MIF. We found that MIF does indeed improve *V*_OC_ and maintains high *J*_SC_ values, the latter being explained by the device morphology and DPP(TBFu)_2_ crystallinity, which are very similar to DPP(TBFu)_2_:PC_61_BM, as evidenced by atomic force microscopy (AFM) and X-ray diffraction (XRD).

## Results

**Figure 2:**
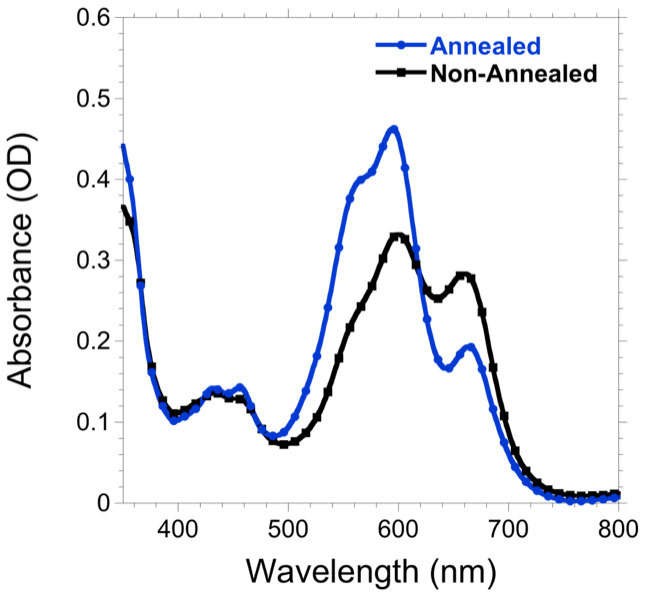
Absorption spectra of non-annealed (black) and annealed (blue) DPP(TBFu)_2_:MIF thin films.

Devices were prepared in a similar manner to previous studies with the following architecture: ITO/PEDOT:PSS/DPP(TBFu)_2_:Fullerene/LiF/Al^26^,^28^. A detailed description of device fabrication is provided in the methods section. The active layers were prepared from a 20 mg/ml solution with DPP(TBFu)_2_:fullerene ratios between 2:3 and 3:2. As in previous reports CHCl_3_ was the solvent of choice for DPP(TBFu)_2_:PC_61_BM^27^,^28^. However, a mixed solvent of CHCl_3_ and chlorobenzene (CB) was required for DPP(TBFu)_2_:MIF to sufficiently dissolve MIF, as it has low solubility in CHCl_3_ (1.5 wt%, see ESI for information on the solubility of MIF in various organic solvents). The ratio of CHCl_3_:CB was the same as that of DPP:MIF in each respective device, although we obtained similar device performances by adding as little as 10% CB to CHCl_3_ (ESI, Fig. S1). Solvent vapour annealing (SVA) was applied after spin-coating the active layer by introducing the substrates into a CH_2_Cl_2_ saturated container for 2 min. SVA is a proven method for producing highly crystalline donor domains for several small molecule donors and allows high fill factor (FF) values to be obtained^26^,^28^,^29^,^30^,^31^. Fig. 2 shows the UV-vis absorption spectra of DPP(TBFu)_2_:MIF films before and after the SVA step. After annealing a characteristic blue shift in the absorption spectrum was observed and is attributed to the aggregation and growth of DPP crystallites within the active layer^28^.

**Figure 3:**
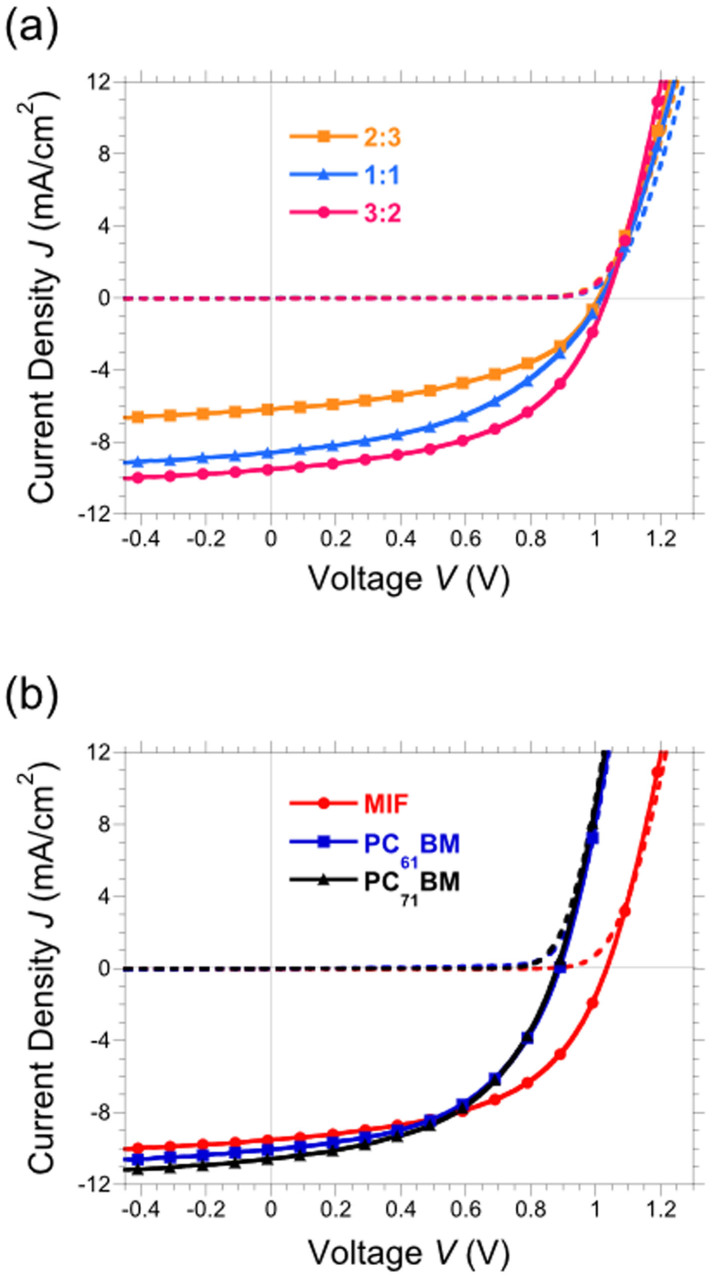
*J-V* characteristics measured under standard 1 sun conditions (AM 1.5 G, 100 mW/cm^2^) (solid lines) and dark conditions (dashed lines) for (a) DPP(TBFu)_2_:MIF devices with the corresponding DPP(TBFu)_2_:MIF ratios: 2:3 (orange, squares), 1:1 (blue, triangles) and 3:2 (red, circles) and (b) 3:2 DPP(TBFu)_2_:fullerene devices, where the fullerene is MIF (red, circles), PC_61_BM (blue, squares) or PC_71_BM (black, triangles).

*J-V* characteristics of DPP(TBFu)_2_:MIF with ratios of 2:3, 1:1 and 3:2 recorded under 1 sun simulated illumination (100 mW/cm^2^, AM 1.5G) and in the dark are shown in Fig. 3a and the figures of merit are presented in Table 1. A donor-acceptor ratio of 3:2 for DPP(TBFu)_2_:MIF was found to be optimum, providing for the higher *J*_SC_ and improved series and shunt resistances (R_S_ and R_P_, respectively), which led to improved FF values; the same ratio was also found to be optimum for both DPP(TBFu)_2_:PCBM devices in agreement with previous studies (see Table 1)^27^,^28^. All devices showed a *V*_OC_ exceeding 1 V, the highest *V*_OC_ being obtained using the 3:2 D:A ratio (1.03 V). Fig. 3b shows *J-V* curves of the best DPP:MIF device alongside reference DPP(TBFu)_2_:PC_61_BM and DPP(TBFu)_2_:PC_71_BM devices fabricated under the same conditions and D:A ratio to allow for the direct comparison between an efficient 58-π fullerene and for comparing with previous literature reports that utilised PC_71_BM^27^,^28^, respectively. We observe that the *V*_OC_ for the 3:2 device is 140 mV higher than the DPP(TBFu)_2_:PC_61_BM and shows only a small decrease in *J*_SC_ (9.52 mA/cm^2^ *vs.* 10.05 mA/cm^2^), while the FF is higher for the MIF based device (0.52 vs 0.50). DPP(TBFu)_2_:PC_71_BM have a similar *V*_OC_ and FF to devices with the C_60_ analogue but have a higher *J*_SC_, as expected due to the better absorption characteristics of C_70_. In terms of efficiency, DPP(TBFu)_2_:MIF devices have a higher efficiency (5.1%) than both DPP(TBFu)_2_:PC_61_BM (4.5%), and DPP(TBFu)_2_:PC_71_BM (4.7%), due to the improved *V*_OC_, which arises from the increased energy difference between the LUMO of MIF and the HOMO of DPP(TBFu)_2_. MIF devices with a 3:2 ratio also have slightly higher FF values, in part due to the increased R_P_. However, the shape of the *J-V* curve can also be affected by geminate and non-geminate recombination^32^. Table 1 provides the figures of merit for all devices presented. Importantly, for this study and in the broader context, the optimum 3:2 DPP(TBFu)_2_:MIF device only shows a slight decrease in *J*_SC_ with respect to DPP(TBFu)_2_:PC_61_BM devices while having a *V*_OC_ over 1 V. The space charge limited current derived hole mobility of 3:2 DPP(TBF)_2_:MIF hole-only devices, 3.1 × 10^−5^ cm^2^ V^−1^ s^−1^, compared well with the equivalent PC_61_BM devices, 2.5 × 10^−5^ cm^2^ V^−1^ s^−1^, which is consistent with the high *J*_SC_ values obtained for the MIF devices.

**Figure 4:**
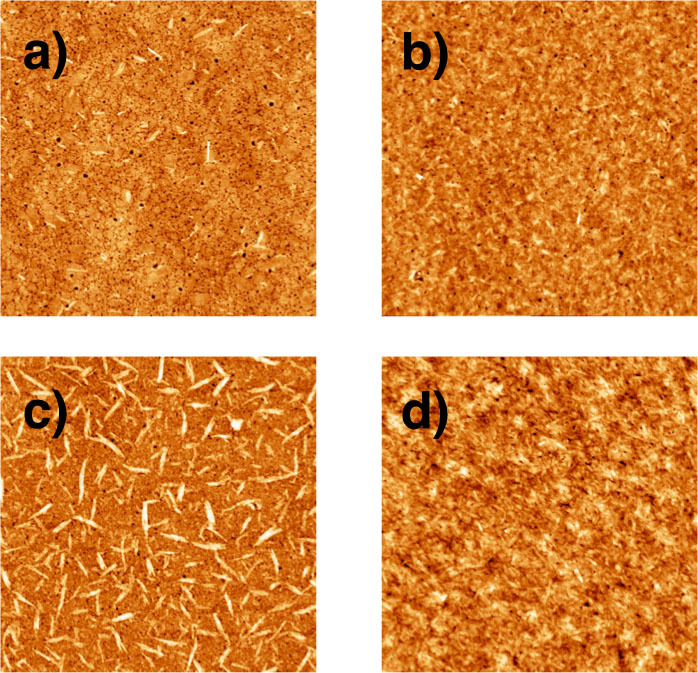
AFM images (5 × 5 μm) of 3:2 DPP(TBFu)_2_:MIF films (a,b) and DPP(TBFu)_2_:PC_61_BM (c,d) before (a,c) and after (b,d) solvent vapour annealing.

Controlling the morphology of BHJ films is key to obtaining high efficiency devices and has been well exemplified in previous DPP(TBFu)_2_ studies^26^,^28^,^33^,^34^,^35^. Poor compatibility between donor and acceptor molecules, which can be identified by imaging non-annealed films, will prevent the formation of a well-ordered BHJ that is necessary for efficient charge generation, separation and extraction^26^. We thus investigated the morphology and crystallinity of DPP(TBFu)_2_:MIF BHJ films and compared them to DPP(TBFu)_2_:PC_61_BM. AFM images of annealed and non-annealed DPP(TBFu)_2_:MIF films are shown in Fig. 4 that demonstrate the good film formation between DPP(TBFu)_2_ and MIF. Non-annealed films show excellent miscibility between both molecules (Fig. 4a) and produce quite flat films (rms roughness = 0.88 nm) with almost no sign of aggregation. Subjecting the active layer to 2 min of SVA in a saturated CH_2_Cl_2_ environment led to a roughening of the surface (rms roughness = 1.25 nm) due to crystallization of DPP(TBFu)_2_ (Fig. 4b), which was observed using out-of-plane X-ray diffraction on the active layers (*vide infra*). The corresponding images of DPP:PC_61_BM are shown in Fig. 4c and 4d. One significant difference to note between the morphology of both blends is that the as-cast DPP(TBFu)_2_:PC_61_BM film shows considerable aggregation compared to DPP(TBFu)_2_:MIF and thus a much rougher surface (rms roughness = 2.04 nm). SVA allows the molecules to re-orientate and the resulting morphology shows no signs of aggregation and the roughness increases slightly (rms roughness = 2.17 nm) due to the more crystalline nature of the films as opposed to aggregates.

**Figure 5:**
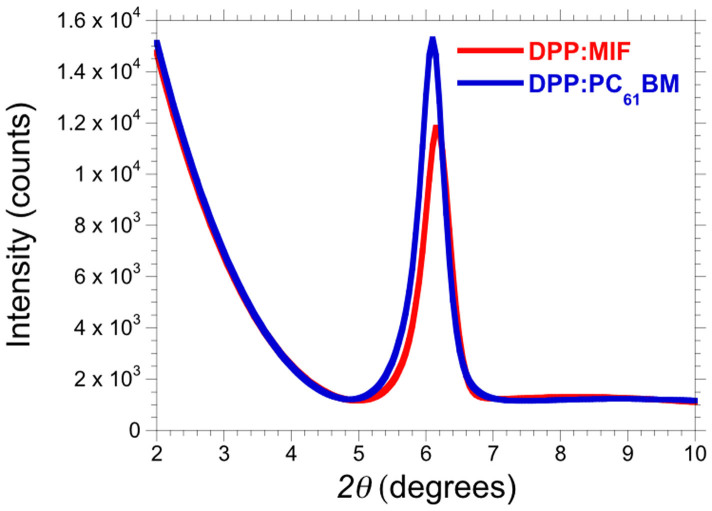
Out of plane XRD diffractograms of SVA treated DPP(TBFu)_2_:MIF and DPP(TBFu)_2_:PC_61_BM thin films on ITO/PEDOT:PSS.

The XRD diffractograms of the DPP:MIF and DPP:PC_61_BM films are shown in Fig. 5 and show a peak at 2*θ* = 6.19° and 6.09°, respectively, corresponding to an inter-plane spacing of 14.3 Å and 14.5 Å, respectively, which is characteristic of pure DPP(TBFu)_2_, as seen in previous studies^27^,^28^. The average crystallite size for DPP(TBFu)_2_ in the annealed DPP(TBFu)_2_:MIF film, calculated using the Scherrer equation, was 17.9 nm^36^, which is very similar to what we calculated for DPP(TBFu)_2_ blended with PC_61_BM, 17.8 nm. There was, however, a difference in the overall crystallite volume observed between DPP(TBFu)_2_:MIF and DPP(TBFu)_2_:PC_61_BM films, with the latter having a higher volume (Fig. 5). No peaks corresponding to MIF were observed. The similarities observed between the AFM and XRD data of DPP(TBFu)_2_:MIF and DPP(TBFu)_2_:PC_61_BM BHJ films help explain the high hole mobility and *J*_SC_ observed in optimum DPP(TBFu)_2_:MIF devices, especially if we consider what was observed by Viterisi *et al.* for DPP(TBFu)_2_ blended with DPM fullerene derivatives, for example, where the morphology of the active layer, as well as the crystallinity of DPP(TBFu)_2_, was significantly affected by the DPM fullerene^26^.

## Discussion

Putting these results into the perspective of previous reports of polymer:fullerene OSCs, very few fullerenes, in particular fullerenes with higher lying LUMO levels, seem to work well with polymers having a lower band-gap than P3HT. The LUMO and HOMO offsets may indeed explain some of the poorer performances observed but the miscibility between donor and acceptor and crystalline properties (both individual and collective) are also key factors that define the device performance. Many efficient low-bandgap polymers for example form amorphous domains, which may prevent optimum charge transport, as already suggested by Faist *et al*.^21^ DPP(TBFu)_2_ has a lower band-gap than P3HT and lower lying frontier molecular orbitals yet still maintains high *J*_SC_ when MIF is the acceptor, meaning that energetic offsets between HOMO-HOMO and LUMO-LUMO levels at the donor-acceptor interface must be sufficiently large so as not to affect charge separation. The domain size of DPP(TBFu)_2_ must also be close to optimum in these devices to allow a high number of excitons to reach the D-A interface, and indeed seems to be, with the average crystallite size being almost identical to that of DPP(TBFu)_2_:PC_61_BM devices. The total crystalline volume of the donor is, however, lower in DPP(TBFu)_2_:MIF active layers, which may explain the slight decrease in *J*_SC_. Furthermore, the AFM topography images show good intermixing of DPP(TBFu)_2_ and MIF and is again similar to what we observe for DPP(TBFu)_2_:PC_61_BM. Moving on from this study, it will be interesting to expand the application of MIF with other low bandgap small molecule and polymeric donors in order to gain a deeper understanding of the key factors that define high performance donor:fullerene BHJ solar cells.

In conclusion, we have applied MIF, a 56-π fullerene containing indene and methanediyl adducts, with a small molecule donor (DPP(TBFu)_2_) for the first time and obtained a power conversion efficiency of 5.1%. Reference DPP(TBFu)_2_:PC_61_BM and DPP(TBFu)_2_:PC_71_BM devices show lower performances of 4.5% and 4.7%, respectively. The higher efficiency of DPP(TBFu)_2_:MIF arises from the higher *V*_OC_ achieved, which stems from MIF's higher lying LUMO level. Furthermore, no considerable changes in *J*_SC_ or FF are observed between MIF and the PCBM reference devices, which can be understood by the excellent miscibility between DPP and MIF and the ability for DPP(TBFu)_2_ to form well ordered crystalline domains during SVA of the BHJ, as observed by AFM and XRD. The results obtained in this study provide key insights into the importance of optimizing both the electronic and molecular structure of fullerene derivatives to allow higher *V*_OC_ values to be obtained without impacting upon the morphology of the donor molecule, which can limit the *J*_SC_.

## Methods

### General notes

DPP(TBFu)_2_ was purchased from Lumtec and further purified by silica gel column chromatography using a toluene:hexane eluent (9:1). MIF was synthesized and purified following our previously reported method^9^. Briefly, under a nitrogen atmosphere, a solution of C_60_(CH_2_) (300 mg, 0.409 mmol) and indene (1.14 mL, 9.81 mmol) in 1,2-dichlorobenzene (60 mL) was refluxed for 20 h. After removal of solvent by vacuum distillation, the obtained solid was subjected to silica gel column chromatography (eluent, CS_2_/hexane = 1/2 to 1/0) to remove starting materials. Gel permeation chromatography was then carried out to isolate MIF.

### Device Fabrication

Devices were fabricated with the architecture: ITO/PEDOT:PSS/DPP(TBFu)_2_:Fullerene/LiF/Al. First, patterned indium-doped tin oxide (ITO) substrates (155 nm, 9 Ω/□) were sonicated in acetone for 15 min followed by two additional 15 min sonication cycles in isopropanol. Next, the substrates were dried under a stream of nitrogen and then subjected to 20 min UV/O_3_ treatment. PEDOT:PSS (Clevios AI4083) was spin-coated onto the clean ITO substrates at a rate of 3000 rpm for 30 sec. Annealing of PEDOT:PSS films was first done in air at 120°C and then in a N_2_ filled glovebox at 130°C for an additional 5 min. Active layers were then deposited by spin-coating at a rate of 3000 rpm for 60 sec. The donor:acceptor ratio (wt:wt) for DPP(TBFu)_2_:MIF devices varied between 2:3 and 3:2 for optimization purposes and had a total concentration of 20 mg/ml in CHCl_3_:chlorobenzene (CB) solution, where the ratio of each solvent (vol:vol) mirrored that of the active layer, *i.e.* 2:3 MIF:DPP(TBFu)_2_ was dissolved in a 2:3 CHCl_3_:CB solution. CB was required for dissolving MIF, however was not necessary for the PCBM reference devices. DPP(TBFu)_2_:PC_61_BM and DPP(TBFu)_2_:PC_71_BM devices had a ratio of 3:2 and the same concentration as the MIF-based devices (20 mg/ml) in CHCl_3_. Active layer thickness was approx. 90 nm as measured using a step-profiler. Solvent vapour annealing (SVA)^14^, which consisted of placing one substrate at a time in a sealed vessel saturated with CH_2_Cl_2_ for 2 min, was applied to each active layer after spin-coating. Following SVA the substrates were placed in an evaporator chamber where a 0.8 nm layer of LiF was first deposited followed by a 100 nm layer of Al. The pressure of the evaporation chamber never exceeded 5 × 10^−5^ mbar during deposition. Devices were sealed in a N_2_ rich environment using a UV curable epoxy before measuring their photovoltaic characteristics. Hole-only devices were fabricated in the same manner as normal devices except that a MoO_3_(8 nm)/Au (100 nm) top electrode was used.

### Device Characterization

Current-voltage (*J-V*) characteristics were measured by software-controlled source meter (Keithley 2400) in dark conditions and 1 sun AM 1.5 G simulated sunlight irradiation (100 mW/cm^2^) using a solar simulator (EMS-35AAA, Ushio Spax Inc.), which was calibrated using a silicon diode (BS-520BK, Bunkokeiki).

### Film Characterization

UV-visible absorption spectra were measured on JASCO V-670 spectrometer (Nihon bunko). Atomic force microscopy images were recorded using a Bruker Multimode atomic force microscope operating in tapping mode (Si probes, nominal frequency 70 kHz). Out-of-plane X-ray diffraction was carried out on a Rigaku Smartlab diffractometer using Cu-Kα radiation operating with a power of 9 kW (45 kV, 200 mA). The diffraction pattern of each sample was recorded between an angular 2*θ* of 2 and 14° at 0.5° increments, the duration of which were 3 sec.

## Author Contributions

J.W.R. and Y.M. conceived the work. J.W.R. designed and performed all experiments and wrote the manuscript. Y.M. designed the concept to have high open-circuit voltage, and modified the manuscript. Both authors discussed and agreed on the final manuscript.

## Supplementary Material

Supplementary Information Supplementary Information

## Figures and Tables

**Figure 1:**
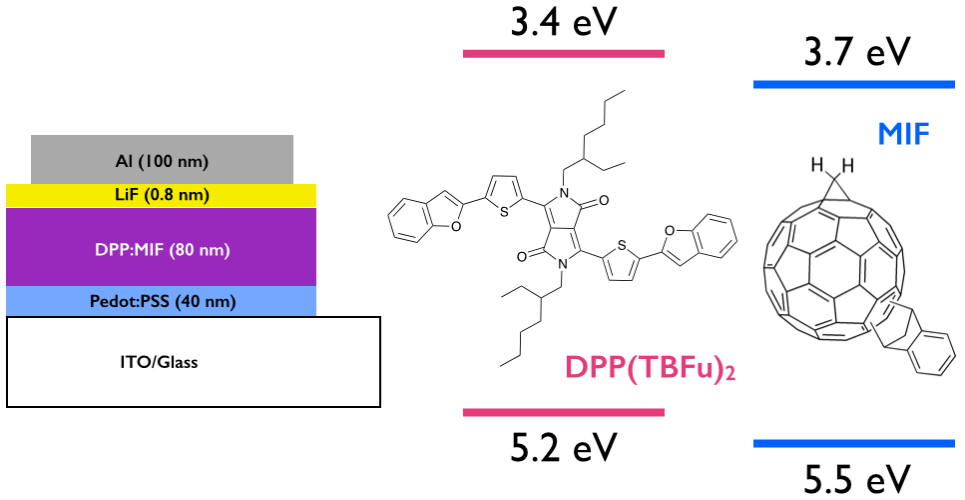
Device architecture and molecular structure of DPP and MIF together with their HOMO-LUMO levels.

**Table 1:** *J-V* characteristics for DPP(TBFu)_2_:MIF (various ratios) and DPP(TBFu)_2_:PCBM devices

Device	*V*_OC_ V	*J*_SC_ mA/cm^2^	FF	η %	R_S_ Ω cm^2^	R_P_ Ω cm^2^
DPP:MIF (2:3)	1.01	6.19	0.47	2.93	10.9	1.3 × 10^5^
DPP:MIF (1:1)	1.02	8.59	0.45	3.97	10.6	1.6 × 10^6^
DPP:MIF (3:2)	1.03	9.52	0.52	5.08	8.1	7.7 × 10^6^
DPP:PC_61_BM	0.89	10.05	0.50	4.46	5.4	1.1 × 10^6^
DPP:PC_71_BM	0.89	10.74	0.50	4.69	5.0	3.6 × 10^6^

The data corresponds to the best device in each. Statistical analysis is provided in the ESI.
