# Supplementary material for: Increased Efficiency in Small Molecule Organic Solar Cells Through the Use of a 56-π Electron Acceptor – Methano Indene Fullerene
Source: Sci Rep. 2015 Feb 9;5:8319. doi: 10.1038/srep08319 (PMC4321161; doi:10.1038/srep08319)
Supplement: Supplementary Information [file srep08319-s1.pdf]

Supplementary Information for:

Increased Efficiency in Small Molecule Organic Solar Cells Through the Use of a 56-π Electron Acceptor – Methano Indene Fullerene

James W. Ryan[a], Yutaka Matsuo[a]*

[a] Department of Chemistry, The University of Tokyo, 7-3-1 Hongo, Bunkyo-ku, Tokyo 113-0033, Japan
matsuo@chem.s.u-tokyo.ac.jp

Solubility of Methano Indene Fullerene (MIF) in Common Solvents

Table S1 Solubility of MIF in common organic solvents

Solvent	Solubility (wt %)
Chloroform	1.5
Toluene	4.0
Chlorobenzene	5.0
1,2-dichlorobenzene	6.5

Additional *J-V* Curves and IPCE Data

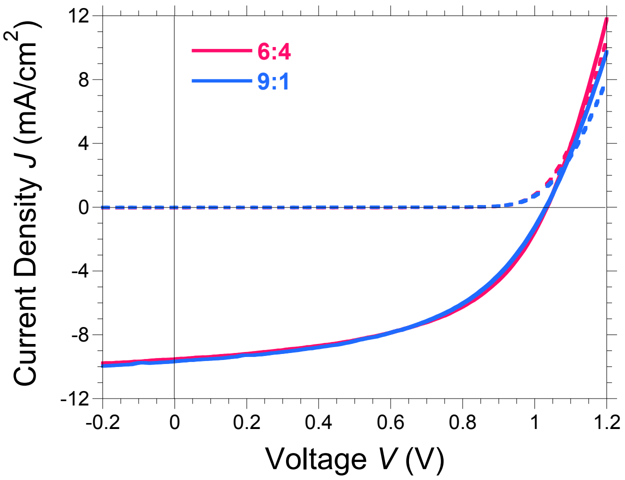

Fig. S1 *J-V* curves in dark (dashed lines) and under 1 sun illumination (solid lines) for 3:2 DPP(TBFu)$_2$:MIF devices using two different $CHCl_3$:chlorobenzene ratios; 6:4 (pink) and 9:1 (blue).

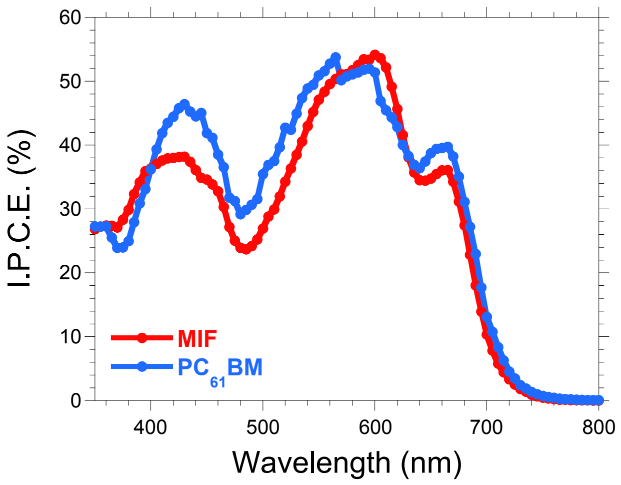

Fig. S2 IPCE spectrum for a 3:2 DPP(TBFu)$_2$:MIF and DPP(TBFu)2:PC$_{61}$BM devices

Note on the *JV* curves in the manuscript. All data presented in the manuscript comes from optimised devices and each device was cross-referenced to ensure the trends presented are accurate. Table S2 shows the mean figures of merit for each device in Table S1 and includes the standard deviation.

Table S2 Mean figures of merit ± standard deviation for devices shown in Table 1 of the manuscript. In each case, the number of devices averaged was 4.

Device	V_{OC} (V)	J_{SC} (mA/cm^2)	FF	η (%)
DPP:MIF (2:3)	0.979 ± 0.055	6.23 ± 0.22	0.47 ± 0.03	2.85 ± 0.14
DPP:MIF (1:1)	1.013 ± 0.015	8.61 ± 0.10	0.45 ± 0.01	3.92 ± 0.12
DPP:MIF (3:2)	1.030 ± 0.004	9.55 ± 0.55	0.51 ± 0.01	5.02 ± 0.09
DPP:PC$_{61}$BM	0.888 ± 0.005	9.73 ± 0.22	0.50 ± 0.00	4.29 ± 0.12
DPP:PC$_{71}$BM	0.876 ± 0.003	10.54 ± 0.13	0.50 ± 0.00	4.58 ± 0.05
